# Synthetic lethality between *ATR* and *POLA1* reveals a potential new target for individualized cancer therapy

**DOI:** 10.1016/j.neo.2024.101038

**Published:** 2024-08-10

**Authors:** Hanna Elisabeth Schneider, Lisa-Maria Schmitt, Albert Job, Brigitte Lankat-Buttgereit, Thomas Gress, Malte Buchholz, Eike Gallmeier

**Affiliations:** a Center for Tumor Biology and Immunology, Department of Gastroenterology, Endocrinology and Metabolism, University Hospital of Marburg, Philipps-University Marburg, Marburg, Germany; b Department of Medicine A - Hematology, Oncology and Pneumology, University Hospital Münster, Muenster, Germany; c Department of Internal Medicine II - Gastroenterology, Oncology and Metabolism, Hospital Memmingen, Memmingen, Germany

**Keywords:** Synthetic letality, ATR, POLA1, Colon cancer, S phase arrest, Apoptosis, Cancer therapy

## Abstract

The ATR-CHK1 pathway plays a fundamental role in the DNA damage response and is therefore an attractive target in cancer therapy. The antitumorous effect of ATR inhibitors is at least partly caused by synthetic lethality between *ATR* and various DNA repair genes. In previous studies, we have identified members of the B-family DNA polymerases as potential lethal partner for *ATR*, i.e. *POLD1* and *PRIM1*. In this study, we validated and characterized the synthetic lethality between *ATR* and *POLA1*.

First, we applied a model of ATR-deficient DLD-1 human colorectal cancer cells to confirm synthetic lethality by using chemical POLA1 inhibition. Analyzing cell cycle and apoptotic markers via FACS and Western blotting, we were able to show that apoptosis and S phase arrest contributed to the increased sensitivity of ATR-deficient cancer cells towards POLA1 inhibitors. Importantly, siRNA-mediated POLA1 depletion in ATR-deficient cells caused similar effects in regard to impaired cell viability and cumulation of apoptotic markers, thus excluding toxic effects of chemical POLA1 inhibition. Conversely, we demonstrated that siRNA-mediated POLA1 depletion sensitized several cancer cell lines towards chemical inhibition of ATR and its main effector kinase CHK1.

In conclusion, the synthetic lethality between *ATR/CHK1* and *POLA1* might represent a novel and promising approach for individualized cancer therapy: First, alterations of POLA1 could serve as a screening parameter for increased sensitivity towards ATR and CHK1 inhibitors. Second, alterations in the ATR-CHK1 pathway might predict in increased sensitivity towards POLA1 inhibitors.

## Graphical abstract

**Figure:**
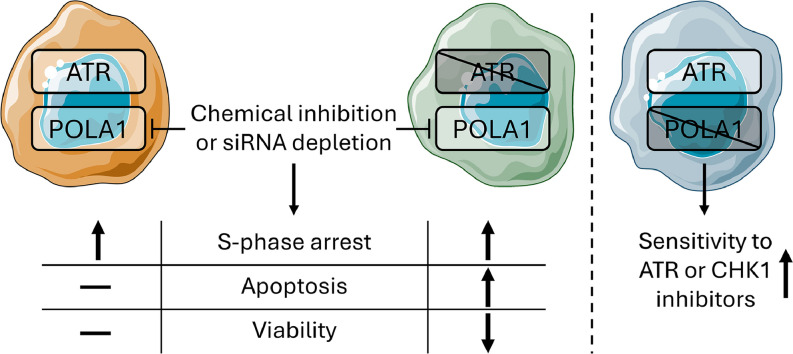

## Introduction

Cancer therapy is one of the most permanent changing subjects of modern medicine. Currently, a main goal is to establish more efficient cancer treatments by using individualized and targeted approaches for tumor cells and thereby to minimize the frequency and severity of side effects. To this end, genotype-based cancer treatment represents a promising tool. During carcinogenesis the accumulation of mutations – particularly within the DNA repair pathways – is one crucial process for the malignant transformation of normal cells to cancer cells. To compensate the impairment in certain DNA repair pathways due to the accumulation of mutations, other DNA repair pathways are often upregulated in cancer cells [20]. This process facilitates novel therapeutic approaches through practical application of the principle of synthetic lethality.

Synthetic lethality describes the relationship between two genes where inactivation of either gene alone has no effect, but inactivation of both genes is lethal to the cell carrying these mutations [32]. Thus, by pharmacologically targeting a gene, which compensates the loss – mediated by mutations during carcinogenesis – of its synthetically lethal partner, synthetic lethality increases toxicity specifically towards cancer cells. A well-known example with clinical impact is the role of poly(ADP-ribose) polymerase (PARP) inhibitors in tumors harboring *BRCA1/2* mutations [15]. This targeted approach is already used for multiple cancer entities including breast, ovarian and pancreatic cancers [26].

One important part of the DNA repair machinery is the phosphoinositide 3-kinase (PIK)-related kinase named ataxia telangiectasia and Rad3-related protein (ATR). Activated by replication protein A (RPA)-coated single stranded DNA (ssDNA), ATR stabilizes the replication fork and mediates cell cycle arrest and DNA repair [6]. ATR exists in a complex with the ATR interacting protein (ATRIP), which enables activation by the topoisomerase binding protein 1 (TopBP1)-RAD9-RAD1-HUS1 (9-1-1) complex [9,30]. Together with claspin, the activated ATR induces cell cycle arrest by phosphorylation of Checkpoint kinase 1 (CHK1), which represents the major downstream effector of ATR [14].

In our previous studies on the synthetically lethal relationship between *ATR* and *POLD1* and *ATR* and *PRIM1* [21,23,24], preliminary experiments indicated another synthetically lethal relationship, namely between *ATR* and *POLA1*, encoding the catalytic subunit of the DNA polymerase α [8]. This assumption was further supported by Rogers et. al. [35], which also confirmed a synthetic lethal relationship between *CHK1* and multiple DNA polymerases such as *POLA1, POLE* and *POLE2*.

To gain a more detailed understanding of the effects already described and thereby provide a basis for clinical benefit, we have investigated and characterized this relationship between *ATR* and *POLA1*. First, we applied a well-described cellular model of ATR-deficiency in the human colorectal cancer cell line DLD-1 [16,21,22,46], consisting of parental DLD-1 cells (subsequently referred to as *ATR^+/+^* cells) and a derived DLD-1 cell clone homozygously harboring the a hypomorphic ATR Seckel mutation (subsequently referred to as *ATR^s/s^* cells). While total loss of ATR is incompatible with cell viability [5], the Seckel mutation applied in this system causes subtotal ATR protein depletion without any detectable effect on viability. To model POLA1 deficiency, we applied ST1926, an atypical retinoid that has already been shown to inhibit POLA1 and proliferation of colorectal cancer cells [1].

Vice versa, as *POLA1* mutations occur in about 7 % of colorectal and pancreatic cancer samples, we additionally used four different ATR and CHK1 inhibitors to assess the chemical inducibility of synthetic lethality in various POLA1-depleted colon and pancreatic cancer cell lines.

Our results illustrate a novel potential approach for individualized cancer therapy using specific ATR/CHK1 inhibitors or POLA1 inhibitors for the individualized treatment of ATR- or POLA1*-*deficient tumors, respectively.

## Material and methods

### Cell culture

Various human colon cancer cell lines (colon: DLD-1 (Leibniz Institut DSMZ, Braunschweig, Germany) and its well characterized *ATR^s/s^* cell clone [16,17,22,46] (Fred Bunz, John Hopkins University, Baltimore, MD, USA), HCT-116 (CLS Cell Line Service GmbH, Eppelheim, Germany), RKO (American Type Culture Collection, LGC Standards, Wesel, Germany); pancreas: PaTu 8988t (Hans-Peter Elsässer, Philipps-University Marburg, Germany)) were cultivated at 37°C and 5 % CO_2_ in Roswell Park Memorial Institute (RPMI 1640) medium or Dulbecco's Modified Eagle Medium (DMEM) (Thermo Fisher Scientific, Schwerte, Germany) containing 10 % fetal bovine serum (FBS) (Thermo Fisher Scientific, Schwerte, Germany).

### Drugs

The following drugs were used while performing experiments: AZD6738 and VE-822 (MedKoo Biosciences, Morrisville, NC), MK-8776 and LY2603618 (Selleckchem, Munich, Germany), and mitomycin C (MMC) and ST1926 (Sigma-Aldrich, Hamburg, Germany).

### Transfection

Protein knockdown was achieved by transfection experiments. For this purpose, HiPerFect (QIAGEN, Hilden, Germany) was mixed with 5 nM siRNA targeting POLA1 (ATGGCAGTCTTTCCTCTCTTA) (QIAGEN, Hilden, Germany) in medium without FBS. After 20 minutes of incubation, this mixture was added to recently plated cells. Allstars negative (non-targeting) and medium without FBS were established as controls to monitor off-target effects.

### Cell proliferations assays

Drug sensitivity was assessed by cell proliferation assays in ATR^+/+^, ATR^+/s^ and ATR^s/s^ cells. Therefore, 1500 cells of ATR^+/+^, ATR^+/s^ and ATR^s/s^ were sown in 96-well plates. After 24 h, they were exposed to the above-mentioned drugs for 120 h. 0.2 % SYBR-green (Lonza, Cologne, Germany) solution in Aqua destillata was added and fluorescence was measured by Victor^3^ V plate reader (PerkinElmer, Waltham, MA). Survival fraction was determined by dividing treated cells by an untreated control group.

### Cell viability assays

Cell viability assays examined either the impact of POLA1 depletion through transfection or the sensibility towards various drugs. For the former, 12,500 – 50,000 ATR^+/+^ cells and 15,000 – 60,000 ATR^s/s^ cells were transferred in 6-well plates, transfected as described above and incubated for 72 – 168 h. For the latter, 140,000 ATR^+/+^, RKO, HCT-116 or PaTu8988t cells were transferred in 6-well plates and transfected as described above. After 72 h, 2,000 – 25,000 cells of POLA1 depleted cells and control samples were plated in 96-well plates. After another 24 h, they were treated with various drugs for 120 h.

Afterwards, cells were washed with Dulbecco´s Phosphate Buffered Saline (DPBS) (Thermo Fisher Scientific, Schwerte, Germany) and treated with MTT. For the MTT-reagent, 0.5 % Thiazolylblue (Roth, Karlsruhe, Germany) solution in DPBS was added to RPMI without FBS. This reagent was added to each well and incubated for 90 minutes. Absorption was measured using a Multiscan FC (Thermo Fisher Scientific, Schwerte, Germany), and cell viability was determined by dividing treated cells by an untreated control group.

### Western blotting

Western blotting was performed as in previous works of our group [23,24]. Protein extracts were extracted by lysing cells, and were then boiled and loaded onto 10 % or 15 % polyacrylamide gels for electrophoresis. Proteins were then transferred to PVDF membranes. Membranes were incubated for 1 h in 5 % milk powder in TBS + 0.1 % Tween 20 (TBS-T) and then incubated overnight with the primary antibody in TBS-T at 4°C. The primary antibodies that were used were anti‐Caspase 3, anti‐cleaved Caspase 3 (Asp175), anti‐Caspase 8 (1C12), anti-PARP and anti-posphoCHK1 (anti-pCHK1; Ser345) (133D3) (Cell Signaling Technology, Cambridge, UK), anti-ATR (N-19), anti‐Caspase 9 (H-170), anti-Cdc25A (5H51), anti-CHK1 (G4), anti‐Cyclin A (H-432), and anti-POLA1 (D-4) (Santa Cruz Biotechnology, Dallas, TX); and anti‐β-Actin (AC-15) (Sigma-Aldrich, Hamburg, Germany). After washing, the membranes were incubated with the secondary antibodies. The secondary antibodies that were used were HRP-conjugated anti-goat, anti-rat, anti-mouse, and anti-rabbit antibodies (Sigma-Aldrich, Hamburg, Germany). Proteins were visualized via chemiluminescence using PerkinElmer Western Lightning Ultra or Clarity Western ECL Substrate (Bio-Rad Laboratories, Munich, Germany) according to the manufacturer's instructions. ß-Actin was included as a loading control. All western blots are representative of at least three separate experiments.

### Flow cytometry

To investigate cell cycle and apoptosis, flow cytometry was performed according to the methods previously described by our group [23,24]. 62,500 – 250,000 ATR^+/+^ cells and 75,000 – 300,000 ATR^s/s^ cells were seeded in 6-well plates and were then treated with ST1926 24 h after seeding. Cells were harvested after a subsequent 24, 48 and 72 h. For cell cycle analysis, cells were washed and stained with propidium iodide (PI; 0.1 % sodium citrate, 0.1 % Triton X-100, 50 μg/ml propidium iodide) according to previously described protocols [31]. For apoptosis analysis, cells were washed and stained with FITC-conjugated Annexin-V (Biolegend, San Diego, CA) diluted 1:40 in Hanks Balanced Salt solution (HBSS) (Thermo Fisher Scientific, Schwerte, Germany) for 20 minutes at room temperature in the dark. Afterwards, 1 µl propium iodide (1mg/ml, Thermo Fisher Scientific, Schwerte, Germany) was immediately added before measurement.

The BD FACSCanto II from BD Biosciences (San Jose, CA) and the FlowJo v10 software from FlowJo, LLC (Ashland, OR) were used to analyze both cell cycle and apoptosis. For each sample, a minimum of 30,000 gated events were assessed. According to Wlodjowic et al. [47] PI^−^/Annexin V^+^ were interpreted as early apoptotic and PI^+^/Annexin V^+^ cells as late apoptotic cells.

### Statistical analyses

GraphPad Prism 9.5.0 (La Jolla, CA, USA) was used for all statistical analyses. Error bars represent ± SD. A minimum of three separately experiments were performed. Statistical analysis was performed by two-way ANOVA with Bonferroni post-hoc test, where P values of P < 0.05 (*), P < 0.01 (**) or P < 0.001 (***) were considered significant.

## Results

### Synthetic lethality between *ATR* and *POLA1* in an ATR-knock-in DLD-1 model

**Fig. 1:**
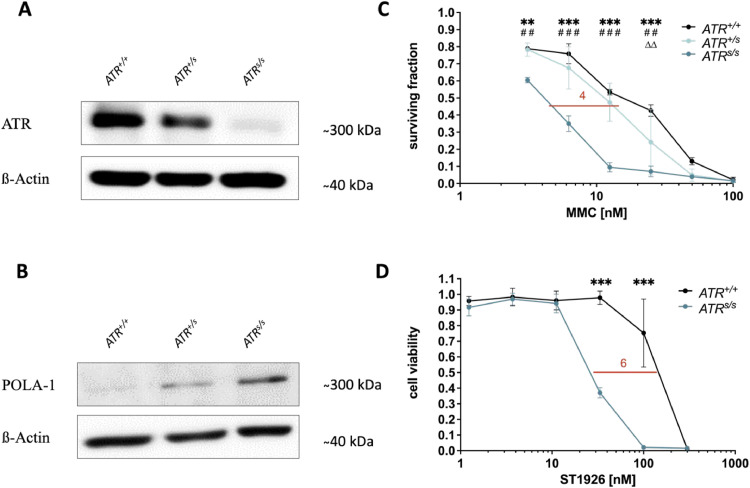
Synthetic lethality between *ATR* and *POLA1* in an ATR-knock-in DLD-1 model. (A) Confirmation of ATR expression in *ATR^+/+^, ATR*^+/s^ and *ATR^s/s^* cells via Western blotting. (B) Confirmation of POLA1 expression in *ATR^+/+^, ATR^+/s^* and *ATR^s/s^* cells via Western blotting. (C) Assessment of MMC sensitivity on the proliferation of *ATR^+/+^, ATR^+/s^* and *ATR^s/s^* cells via proliferation assay 120 h post treatment. (D) Assessment of ST1926 sensitivity on the proliferation of *ATR^+/+^* and *ATR^s/s^* cells via MTT-assay 120 h post treatment. Data points are based on triplicate wells of a minimum of three separate experiments. Error bars represent ± SD. Statistical analysis was performed by two-way ANOVA with Bonferroni post-hoc test, where P values of P < 0.05 (*), P < 0.01 (**) or P < 0.001 (***) were considered significant. Asterisks were used for comparisons between *ATR^+/+^* and ATR^s/s^, triangles for comparisons between *ATR^+/+^* and *ATR^+/s^*, hash symbols for comparisons between *ATR^+/s^* and *ATR^s/s^*. Western blots show representative results of at least three separately performed experiments.

To verify the synthetic lethal interaction between *ATR* and *POLA1* we initially applied a well-described model of ATR-deficient DLD-1 human colorectal cancer cells [16,21,22,46]. First, we confirmed and quantified ATR and POLA1 expression in these cell lines via Western blotting (Fig. 1A and B). In concordance with our previous studies, *ATR^+/+^* cells showed the highest ATR protein levels, whereas the ATR levels slightly decreased in *ATR^+/s^* cells and were almost undetectable in *ATR^s/s^* cells (Fig. 1A). Interestingly, POLA1 protein levels reciprocally increased with decreasing ATR protein levels, showing the highest expression in *ATR^s/s^* cells and the lowest expression in *ATR^+/+^* cells (Fig. 1B). Next, we confirmed the previously described increased sensitivity of *ATR^s/s^* cells towards the DNA interstrand-crosslinking agent MMC [17] as compared to *ATR^+/+^* and *ATR^+/s^* cells (Fig. 1C). Then, we assessed the sensitivity of *ATR^s/s^* as compared to *ATR^+/+^* cells towards chemical POLA1 inhibition using ST1926 (Fig. 1D). Notably, with IC_50_ ratios of 6 and 4, respectively, ST1926 showed at least comparable if not even stronger effects than MMC. Taken together, these data illustrate a synthetically lethal relationship between *ATR* and *POLA1* through simultaneous impairment of *ATR* and *POLA1* in DLD-1 colorectal cancer cells*.*

### ST1926-mediated effects on cell cycle distribution in *ATR^+/+^* cells vs *ATR^s/s^* cells

**Fig. 2:**
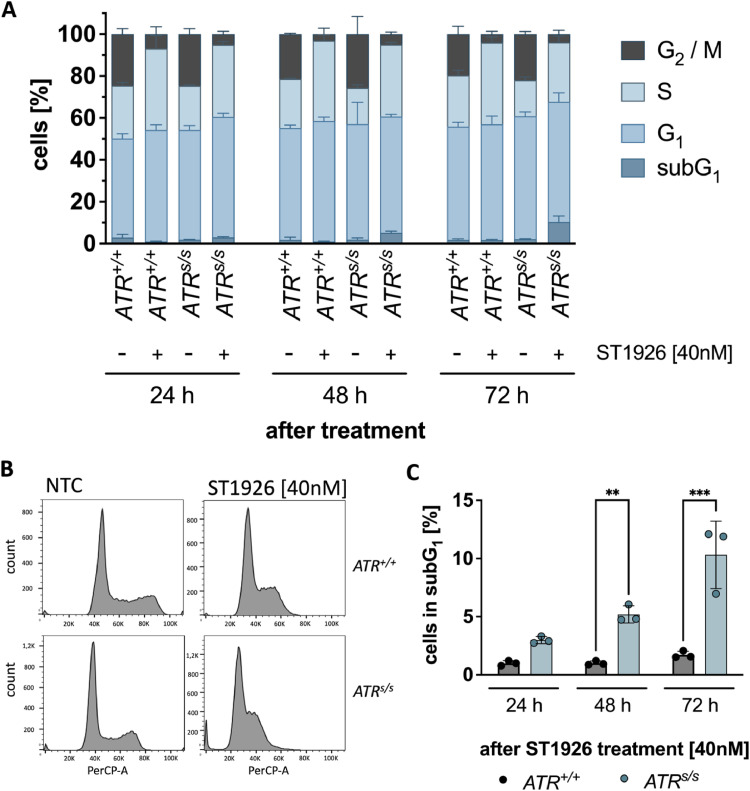
ST1926-mediated effects on cell cycle distribution in *ATR^+/+^* cells vs *ATR^s/s^* cells. (A) Cell cycle profile of *ATR^+/+^* and *ATR^s/s^* cells 24 h, 48 h and 72 h after ST1926 treatment assessed by FACS analysis. (B) Histograms of *ATR^+/+^* and *ATR^s/s^* cells 48 h after ST1926 [40nM] treatment as measured by FACS analysis. (C) SubG_1_ fraction of the cell cycle analysis (Fig. 2A) as measured by FACS analysis**.** Data points are based on triplicate wells of a minimum of three separate experiments. Error bars represent ±SD. Statistical analysis was performed by two-way ANOVA with Bonferroni post-hoc test, where P values of P < 0.05 (*), P < 0.01 (**) or P < 0.001 (***) were considered significant.

To assess whether cell cycle arrest or apoptosis contributed to the increased sensitivity of *ATR^s/s^* cells towards ST1926, we next examined the cell cycle distribution including the sub-G_1_ fraction in *ATR^+/+^* cells and *ATR^s/s^* cells 24 h, 48 h and 72 h after ST1926 treatment. Upon treatment with ST1926, both *ATR^+/+^* and *ATR^s/s^* cells showed increased S phase- and decreased G_2_/M phase-fractions (Fig. 2A). In contrast, only *ATR^s/s^* cells but not *ATR^+/+^* cells showed a significantly increased sub-G_1_ fraction upon ST1926 treatment (Fig. 2B). This effect increased in a time-dependent manner with approximately 10 % of cells in sub-G_1_ at 72 h post treatment (Fig. 2C). Thus, the POLA1 inhibitor ST1926 induced S phase-arrest in both ATR-proficient and ATR-deficient DLD-1 cells, but consecutive apoptosis (as indicated by the subG_1_ fraction) only and specifically in cells lacking ATR.

### ST1926-mediated induction of apoptosis in *ATR^+/+^* cells vs *ATR^s/s^* cells

**Fig. 3:**
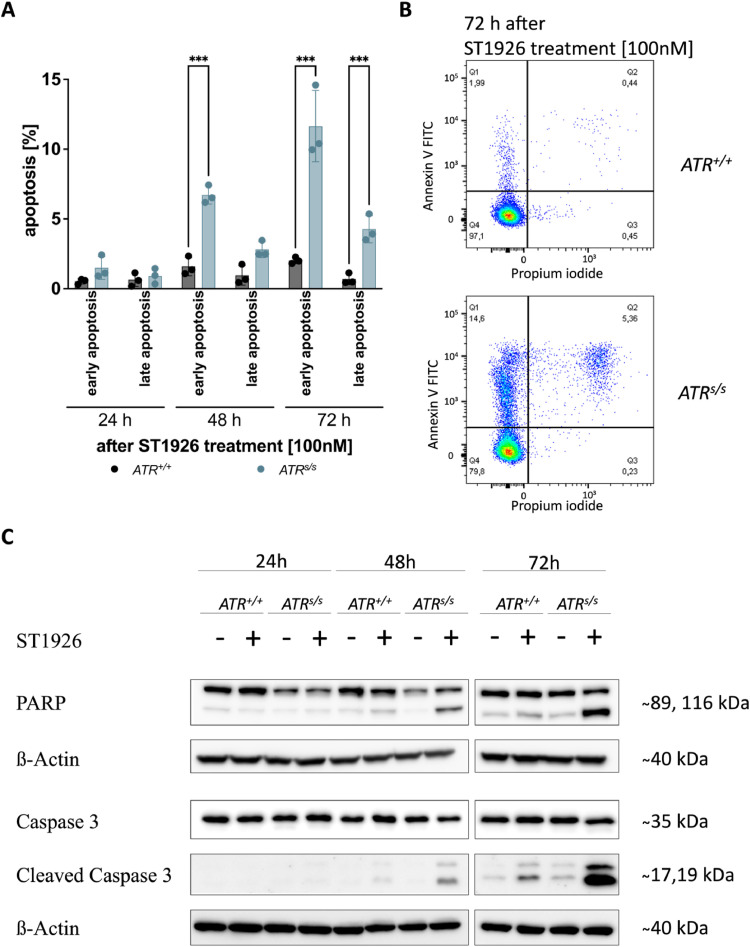
ST1926-mediated induction of apoptosis in *ATR^+/+^* cells vs *ATR^s/s^* cells. (A) Quantification of early apoptotic (PI^−^/annexin V^+^) and late apoptotic cells (PI^+^/annexin V^+^) in *ATR^+/+^* and *ATR^s/s^* cells at 24, 48 and 72 h post treatment with 100 nM ST1926 as measured by flow cytometry. (B) Representative histograms of data shown in (A) of *ATR^+/+^* and *ATR^s/s^* cells 72 h post treatment with 100 nM ST1926 as measured by flow cytometry. (C) Representative results of essential apoptotic markers in *ATR^+/+^* and *ATR^s/s^* cells by Western blotting 24, 48 and 72 h post treatment with 100 nM ST1926. Data points are based on triplicate wells of a minimum of three separate experiments. Error bars represent ± SD. Statistical analysis was performed by two-way ANOVA with Bonferroni post-hoc test, where P values of P < 0.05 (*), P < 0.01 (**) or P < 0.001 (***) were considered significant.

To evaluate whether the observed increased sub-G_1_ fraction in *ATR^s/s^* cells was indeed ascribable to apoptosis, we next performed Annexin V assays in *ATR^+/+^* cells and *ATR^s/s^* cells upon ST1926 treatment. While neither *ATR^+/+^* cells nor *ATR^s/s^* cells showed an increase of Annexin V^+^ without ST1926 treatment (data not shown), we observed an increase of early (PI^−^/Annexin V^+^) as well as late apoptosis (PI^+^/Annexin V^+^) in *ATR^s/s^* cells as compared to *ATR^+/+^* cells upon ST1926 treatment (Fig. 3A and B). While this effect was not yet visible after 24 h, the percentage of Annexin V^+^ *ATR^s/s^* cells increased continuously over the following time points. To characterize apoptosis more specifically and mechanistically, we examined the protein levels of essential apoptosis markers via Western blotting in *ATR^s/s^* and *ATR^+/+^* cells 24 h, 48 h and 72 h post treatment with ST1926 (Fig. 3C). We found an ST1926-induced increase of PARP cleavage exclusively in *ATR^s/s^* cells but not in *ATR^+/+^* cells. Accordingly, the cleavage of caspase 3, the main effector caspase in the execution phase of apoptosis [13], was also increased specifically in *ATR^s/s^* cells at 48 h and 72 h but not in *ATR^+/+^* cells after ST1926 treatment. Taken together, the increased sensitivity of *ATR^s/s^* cells towards chemical POLA1 inhibition can therefore be explained, at least in part, by S-phase arrest and subsequent apoptosis as contributing mechanisms.

### siRNA-mediated POLA1 depletion in *ATR^+/+^* cells vs *ATR^s/s^* cells

**Fig. 4:**
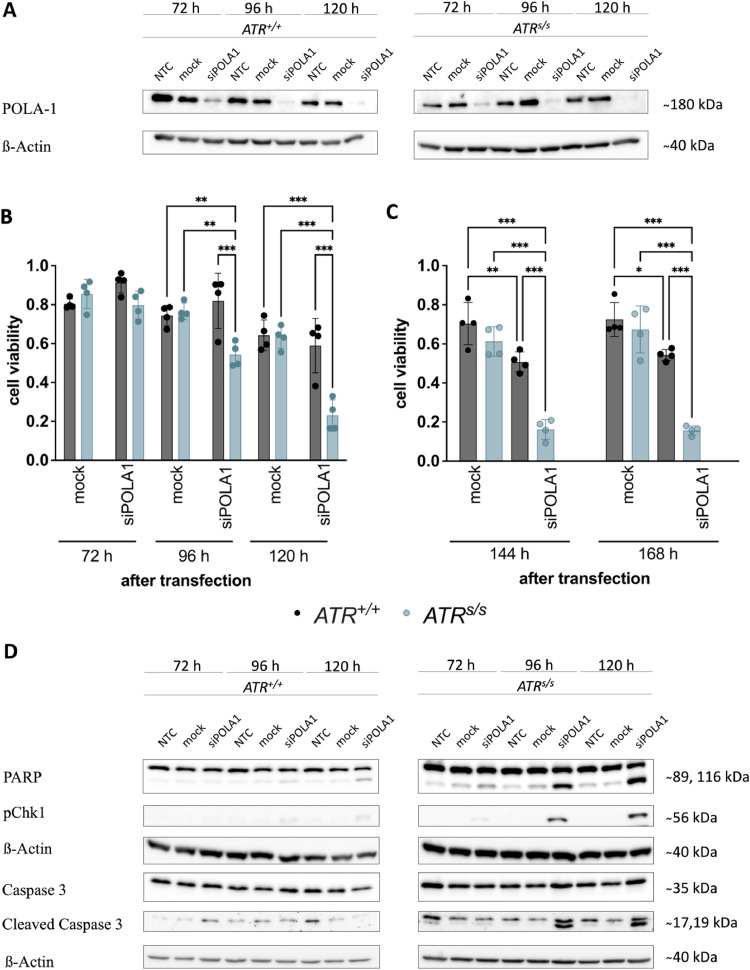
siRNA-mediated POLA1 depletion in *ATR^+/+^* cells vs *ATR^s/s^* cells. (A) Depletion of POLA1 in *ATR^+/+^* and *ATR^s/s^* cells 72, 96 and 120 h after siRNA mediated knock-down shown via Western blotting. A minimum of three independent experiments were performed. (B) Cell viability after siRNA mediated knock-down in *ATR^+/+^* and *ATR^s/s^* cells 72, 96 and 120 h after transfection. (C) Cell viability after siRNA mediated knock-down in *ATR^+/+^* and *ATR^s/s^* cells 144 and 168 h after transfection. Cell viability of siPOLA1- and mock-transfected cells was calculated on the basis of non-treated cells. Data points are based on triplicate wells of a minimum of three separate experiments. Error bars represent ±SD. Statistical analysis was performed by two-way ANOVA with Bonferroni post-hoc test, where P values of P < 0.05 (*), P < 0.01 (**) or P < 0.001 (***) were considered significant. (D) Protein quantification of apoptotic markers in *ATR^+/+^* and *ATR^s/s^* cells at 72, 96 and 120 h after siRNA mediated POLA1 knock-down via Western blotting. A minimum of three independent experiments were performed.

We wanted to examine the effects of a simultaneous inactivation of *ATR* and *POLA1* in another, perhaps more specific model of POLA1 inactivation. Therefore, we tested the effects of POLA1 protein depletion in *ATR^+/+^* cells vs *ATR^s/s^* cells. Efficient POLA1 depletion was confirmed in both *ATR^+/+^* cells and *ATR^s/s^* cells at 72 h, 96 h and 120 h after siPOLA1 transfection (Fig. 4A). We observed an increased sensitivity of *ATR^s/s^* cells 72 – 168 h after siPOLA1 transfection as compared to *ATR^+/+^* cells (Fig. 4B, C). To test whether apoptosis contributed to these effects – as similarly shown for ST1926 – we further quantified protein levels of essential apoptosis markers via Western blotting in *ATR^+/+^* cells vs *ATR^s/s^* cells upon siPOLA1 transfection. Increased levels of cleaved PARP, pChk1 and cleaved Caspase 3 was observed at 96 h and 120 h after siPOLA1 transfection specifically in *ATR^s/s^* cells (Fig. 4D). After siRNA-mediated POLA1 depletion, only the *ATR^s/s^* cells showed increased cleaved caspase 3 levels. Similarly, the levels of Poly(ADP-ribose) polymerase (PARP) were elevated in POLA1 depleted *ATR^s/s^* cells. In addition, CHK1, the major downstream effector kinase of ATR [34,49] was phosphorylated after POLA1 depletion only in *ATR^s/s^*. These effects became apparent at 96 h and lasted until 120 h after transfection. Thus, siRNA-mediated POLA1 depletion induced similar effects as did ST1926 in ATR-deficient cells, further supporting our hypothesis of synthetic lethality between *ATR* and *POLA1* in colorectal cancer cells.

### siRNA-mediated POLA1 depletion sensitizes ATR-proficient DLD-1 cells towards ATR and CHK1 inhibitors

**Fig. 5:**
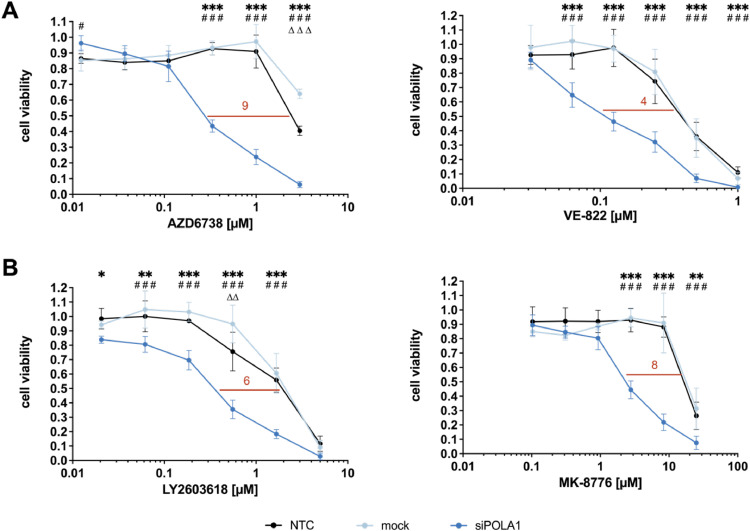
siRNA-mediated POLA1 depletion sensitizes ATR-proficient DLD-1 cells towards ATR and CHK1 inhibitors. Sensitization towards (A) ATR inhibitors and (B) CHK1 inhibitors was measured after 120 h of drug treatment in POLA1*-*depleted *ATR^+/+^* cells vs. control and mock-transfected *ATR^+/+^* cells by MTT-assay. Data points are based on triplicate wells from a minimum of three separate experiments. Error bars represent ± SD. performed. Statistical analysis was performed by two-way ANOVA with Bonferroni post-hoc test, where P values of P < 0.05 (*), P < 0.01 (**) or P < 0.001 (***) were considered significant. Asterisks were used for comparisons between NTC and siPOLA1, triangles for comparisons between NTC and mock, hash symbols for comparisons between mock and siPOLA1.

We next assessed whether - similar to the interactions between ATR and POLA1 observed in the above model systems - ATR or CHK1inhibitors increased the sensitivity of ATR-proficient DLD-1-cells towards siRNA-mediated POLA1 depletion, applying widely used ATR inhibitors (AZD6738 and VE-822) or CHK1 inhibitors (LY2603618 and MK-8776), respectively [34]. As compared to non-transfected cells (NTC) or mock-transfected cells, only siPOLA1-transfected cells showed a significant increased sensitivity towards treatment with AZD6738 and VE-822 (IC_50_ ratios of 9 and 4) (Fig. 5A) and with LY2603618 and MK-8776 (IC_50_ ratios of 6 and 8) (Fig. 5B). Thus, siRNA-mediated POLA1 depletion sensitizes *ATR^+/+^* cells towards chemical ATR- and CHK1-inhibition, respectively.

### siRNA-mediated POLA1 depletion sensitizes HCT-116 and PaTu8988t cells towards ATR and CHK1 inhibitors

**Fig. 6:**
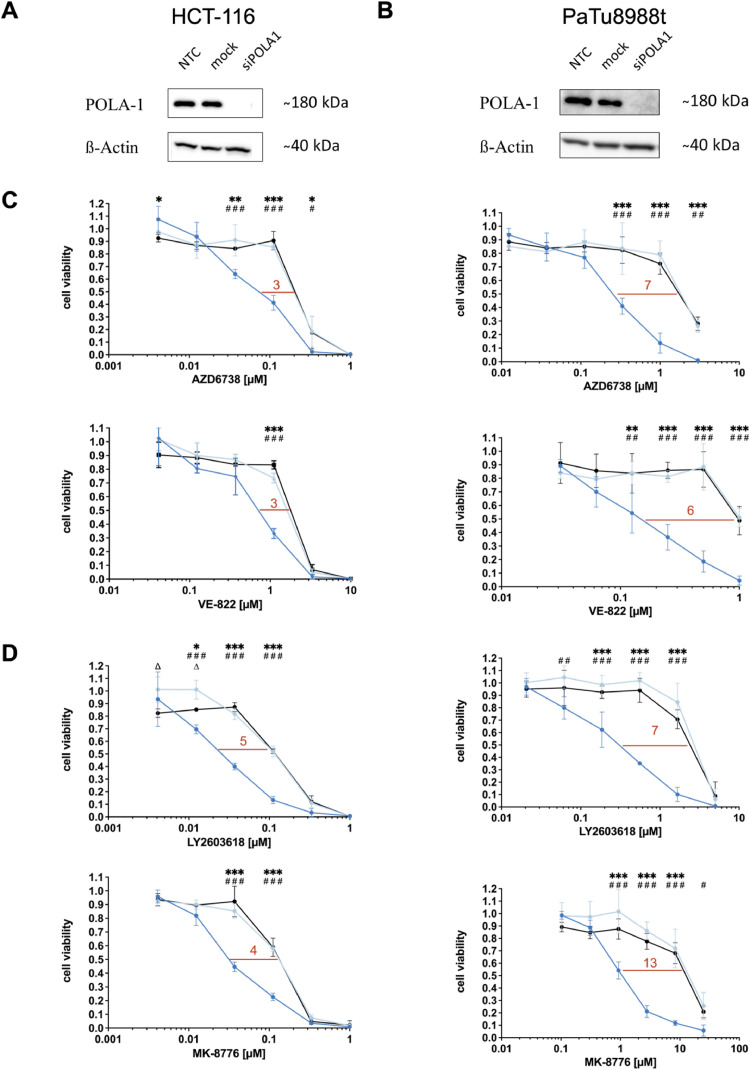
siRNA-mediated POLA1 depletion sensitizes HCT-116 and PaTu8988t cells towards ATR and CHK1 inhibitors. POLA1 depletion after siRNA mediated knock-down in (A) HCT-116 cells and (B) PaTu 8988t cells 72 h after transfection via Western blotting. A minimum of three independent experiments were performed. Sensitization towards (C) ATR inhibitors and (D) CHK1 inhibitors was measured after 120 h of drug treatment in POLA1 depleted HCT-116 or PaTu 8988t cells vs. control and mock-transfected HCT-116 or PaTu 8988t cells by MTT-assay. Data points are based on triplicate wells from a minimum of three separate experiments. Error bars represent ± SD. Statistical analysis was performed by two-way ANOVA with Bonferroni post-hoc test, where P values of P < 0.05 (*), P < 0.01 (**) or P < 0.001 (***) were considered significant. Asterisks were used for comparisons between NTC and siPOLA1, triangles for comparisons between NTC and mock, hash symbols for comparisons between mock and siPOLA1.

To test whether our data initially obtained in DLD-1 were generalizable beyond one cell line or tumor entity, we next assessed whether POLA1 depletion also sensitized other cancer cell lines towards ATR/CHK1-inhibition. Therefore, the colorectal cancer cell line HCT-116 and the pancreatic cancer cell line PaTu8988t were treated with ATR and CHK1 inhibitors, respectively. After verification of efficient POLA1 depletion (Fig. 6A and B), we were able to show a significant increased sensitivity towards treatment with ATR inhibitors AZD6738 and VE-822 upon siPOLA1-transfection in both cell lines (IC_50_ ratios between 3 and 7; Fig. 6C). Similarly, we observed in both cell lines a significant increased sensitivity towards treatment with CHK1 inhibitors LY2603618 and MK-8776 upon siPOLA1-transfection (IC_50_ ratios between 3 and 13; Fig. 6D). Thus, siPOLA1-mediated sensitization to ATR and CHK1 inhibitors is not a cell line-specific phenomenon of DLD-1 cells but can be generalized to a panel of cell lines of different tumor entities.

## Discussion

Using siRNA-library screening of DNA repair genes, we previously identified several genes from B-family DNA-polymerases as potential synthetic lethal partners for the checkpoint kinase ATR of which we consecutively characterized *POLD1* and *PRIM1* in follow-up studies [21,23,24]. In this study, we validated and characterized the relationship between *ATR* and another previously identified candidate partner, i.e. *POLA1*. To this end, we applied various genetic, epigenetic and chemical experimental settings to model ATR- and POLA1-impairment.

In line with our initial hypothesis of synthetic lethality between *POLA1* and *ATR*, we first demonstrated in *ATR^+/+^* and *ATR^s/s^* cells that POLA1 protein levels increased reciprocally with decreasing ATR protein levels, suggesting a perhaps compensatory activation of POLA1 upon inactivation of ATR. This hypothesis was further corroborated by the increased sensitivity of ATR-deficient DLD-1 cells towards treatment with the POLA1 inhibitor ST1926.

To gain more mechanistic insights into the synthetically lethal relationship between *ATR* and *POLA1*, we next investigated whether cell cycle perturbations or apoptosis contributed to the observed effects. ST1926 has already been reported to induce cytostatic and cytotoxic effects including S-phase arrest and apoptosis [[1], [2], [3],^43^]. Besides, apoptosis and cell cycle arrest has also been reported in glioblastoma cell lines treated with ST1926. However, compared to the previously described S phase arrest, this was associated with G0/G1 arrest and a significant reduction of cells in S phase [12]. Interestingly, in our study both ATR-proficient and ATR-deficient cells displayed an increased S and decreased G_2_/M phase upon ST1926 treatment, while increased subG_1_ phase was only detectable in ATR-deficient cells. This could indicate that after ST1926-induced cell cycle arrest only ATR-proficient cells can initiate sufficient DNA damage repair to prevent replication catastrophe, while ATR-deficient cells cannot [41].

ATR is activated by RPA coated ssDNA and suppresses the emergence of more ssDNA and the RPA exhaustion by inhibiting origin firing. Furthermore, it is proficient to prevent replication catastrophe [42]. Interestingly, Ercilla *et. al.* [14] showed that POLA1 activity is also capable to prevent replication catastrophe by avoiding accumulation of ssDNA and RPA exhaustion. Thus, we presume that vice versa the inhibition of POLA1 induces accumulation of ssDNA and consumption of RPA. This assumption is supported by RPA phosphorylation after sole POLA1 depletion, indicating that lower levels of POLA1 might correlate with the amount of replicative stress [35]. If POLA1 is thus inhibited in ATR- or CHK1-deficient cancer cells, global RPA exhaustion and therefore replication catastrophe culminating into apoptosis might be the consequence.

As we observed an increased subG_1_ fraction in ATR-deficient cells upon POLA1 inhibition, we assumed that apoptosis contributed to the synthetically lethal interactions between *ATR* and *POLA1,* which was confirmed by the elevated levels of Annexin V, Caspase 3 cleavage and PARP cleavage in ATR and POLA1 co-depleted cells. Caspase 3 is a central executioner of regulated cell death and is activated by other caspases [18]. PARP cleavage itself is catalyzed by Caspase 3 to prevent depletion of PARP substrates and is therefore also an indicator for apoptosis [4,38]. Thus, the growth inhibition and decreased cell viability induced by the simultaneous ATR and POLA1-impairment can be ascribed at least partially to apoptosis.

Further, POLA1 depletion combined with ATR/CHK1 deficiency induced by chemical ATR/CHK1 inhibition decreased cell viability significantly in our experiments. This phenomenon was observed not only in our initial model of DLD-1 colorectal cancer cells, but was generalizable to various cell lines of different tumor entities. Although POLA1 knockdown was also successfully performed in the colorectal cancer cell line RKO, we did not observe similar effects upon treatment with ATR or CHK1 inhibitors, respectively, in POLA1 depleted cells (data not shown), indicating that expectedly, the therapeutic targeting of POLA1 impaired or vice versa ATR impaired cells using the respective inhibitors will not unequivocally work in all cell lines. This is likely explainable by the highly heterogenous mutation patterns of different tumor cell lines, some of which could harbor mutations that overrule the synthetically lethal effects between *ATR*/C*HK1* and *POLA1.*

Of note, our data demonstrating increased sensitivity of ATR/CHK1 inhibitors in POLA1 depleted cells are strongly supported by already published data [35] showing synthetic lethality between CHK1 and B-family DNA polymerase including POLA1 in both lung and colorectal cancer cells. In that study, it was demonstrated that the simultaneously chemical inhibition of POLA1 and CHK1 increases replication stress, DNA damage and apoptosis compared to single drug using. Some of these B-family DNA polymerase members, e.g. POLD1 and POLE, are already known to be mutated in some familial colorectal carcinomas and adenomas [33]. Although such a correlation is not yet known for *POLA1, POLA1* mutations are found in 6,7 % of tested colon cancer samples and in 22 out of 55 tested colon cancer cell lines according to Catalogue of Somatic Mutations In Cancer and Cell (COSMIC) [10] by Sanger Institute. In addition, studies showed that colorectal cancer cells which are resistant towards ST1926 and its parent molecule CD437 develop *POLA1* mutations [1,19]. Future studies are needed to determine, whether these previously described *POLA1* mutations in other malignant entities are similarly interacting synthetically lethal with ATR and CHK1 inhibitors as has been shown in our study.

The potential impact on clinical anticancer therapy might not only be restricted to pathogenic *POLA1* mutations but potentially also extendable to mere altered POLA1 expression, as low POLA1 expression leads to increased sensitivity against CHK1 inhibitors [35], consistent with high yH2AX levels as an indicator for increased replication stress in CHK1 and POLA1 siRNA-mediated co-depleted cells [40]. Therefore, altered POLA1 expression could potentially also serve as indicator for tumor response after treatment with CHK1 inhibitors.

In addition, Takahashi *et al.* [39] developed a transcriptional profile, the repstress score, for replication stress that includes POLA1 as a marker. The repstress score has been shown to predict sensitivity to ATR inhibitors and is therefore a potential tool for patient selection. Although many other factors are included in this score, it further corroborates the relationship between POLA1 and ATR as characterized in our study.

Finally, *CHK1* frameshift mutations might contribute to tumorgenesis in microsatellite instable colon carcinomas due to consecutive defects in the DNA damage response [27]. Although COSMIC by Sanger Institute only counted approximately 2 % of *CHK1* mutations in the large intestine, many of these alterations could have a pathological impact with almost 30 % frameshift insertions or deletions [10]. Therefore, it would be interesting to analyze the effects of POLA1 inhibitors specifically in cancer cells harboring these mutations. Unfortunately, a Phase I clinical trial in patients with advanced ovarian cancer displayed reduced bioavailability of ST1926 due to glucuroconjugation [36] and was therefore not further investigated. Nevertheless, ST1926 was able to reduce tumor burden and prolong survival in a murine model.

In conclusion, our study suggests that the synthetically lethal effects of simultaneous impairment of ATR and POLA1 in cancer cells might represent a novel and promising approach for individualized cancer therapy. Specific functional *POLA1* mutations could potentially serve as a new biomarker for selection of tumors with an increased sensitivity towards ATR or CHK1 inhibitors and, vice versa, *CHK1* mutations might serve as a new biomarker to predict an increased sensitivity towards POLA1 inhibitors. Currently, novel compounds with POLA1 inhibitory activity are tested in murine xenograft models [7]. Interestingly, two novel dual inhibitors, MIR002 and GEM144, have been shown to specifically inhibit POLA1, causing S-phase arrest and activation of the ATR pathway, and have also demonstrated significant antitumor activity when administered orally in two different human orthotopic malignant pleural mesothelioma xenografts [11]. Moreover, many chemical ATR and CHK1 inhibitors are already investigated in phase I or II clinical trials (e.g. the ATR inhibitor AZD6738 [29,45] and M6620 [48] or CHK1 inhibitors MK-8776 [44], LY2603618 [37] and SRA737 [25,28]). These studies, especially when stratifiable by mutational tumor status in regard to *POLA1, ATR* and *CHK1*, respectively, will further elucidate the potential clinical implications of our study.

## Funding

This work was funded in part by a grant of 10.13039/501100001659Deutsche Forschungsgemeinschaft to E. G. (DFG 762/3-2).

## CRediT authorship contribution statement

**Hanna Elisabeth Schneider:** Writing – original draft, Visualization, Methodology, Investigation, Formal analysis. **Lisa-Maria Schmitt:** Visualization, Investigation. **Albert Job:** Supervision, Methodology. **Brigitte Lankat-Buttgereit:** Supervision, Methodology. **Thomas Gress:** Resources, Funding acquisition. **Malte Buchholz:** Supervision, Resources. **Eike Gallmeier:** Writing – review & editing, Supervision, Resources, Project administration, Methodology, Funding acquisition, Conceptualization.

## Declaration of competing interest

The authors declare that they have no known competing financial interests or personal relationships that could have appeared to influence the work reported in this paper.
